# Clinical factors and practical strategies to optimize amikacin liposome inhalation suspension therapy in refractory *Mycobacterium avium* complex pulmonary disease

**DOI:** 10.1186/s12890-026-04413-w

**Published:** 2026-06-10

**Authors:** Fumio Kurosaki, Ayako Takigami, Ayako Kurosaki, Shu Hisata, Masayuki Nakayama, Naoko Mato, Makoto Maemondo

**Affiliations:** 1 Division of Pulmonary Medicine, Department of Medicine, Jichi Medical University, 3311-1 Yakushiji, Shimotsuke, 329-0498 Tochigi Japan; 2 Department of Palliative Care Medicine, Jichi Medical University, 3311-1 Yakushiji, Shimotsuke, 329-0498 Tochigi Japan

**Keywords:** *Mycobacterium avium* complex, Refractory MAC-PD, Amikacin liposome inhalation suspension, Treatment outcomes, Adherence

## Abstract

**Background:**

Amikacin liposome inhalation suspension (ALIS) has been used in several countries including Japan for refractory *Mycobacterium avium* complex pulmonary disease (MAC-PD). Although its efficacy and adverse events have been reported, factors that limit optimal treatment outcomes in real-world practice are not fully understood. Therefore, we aimed to provide practical considerations for optimizing ALIS therapy by identifying factors associated with culture conversion.

**Methods:**

We retrospectively reviewed the medical records of patients with refractory MAC-PD who were treated with ALIS at a single center between October 2021 and June 2025. Patients treated for ≥ 6 months were included and categorized into culture conversion and non-conversion groups. Clinical characteristics and treatment-related factors were analyzed. Follow-up continued until December 2025.

**Results:**

During the study period, 30 patients with refractory MAC-PD were treated with ALIS. Three patients who discontinued ALIS within 6 months were excluded from further analysis because of MAC-related death (*n* = 1) or severe ALIS-related adverse events (*n* = 2). Consequently, 27 patients were included in the analysis. The median age was 69.9 years, and the median duration of ALIS therapy was 14.6 months. The sputum culture conversion rate was 51.8% (14/27; 95% CI, 34.0-69.3%). Pulmonary fungal disease was identified in 7 patients (25.9%), including newly diagnosed cases during ALIS therapy; however, pulmonary fungal disease was not significantly associated with culture conversion. Cavitary lesions and prior aminoglycoside use were significantly associated with non-conversion. In addition, inappropriate ALIS administration, including prolonged intermittent use and inadequate nebulizer handset replacement, was significantly associated with non-conversion. Dysphonia was the most frequent adverse event and occasionally led to prolonged intermittent use of ALIS. ALIS-related lung abnormalities on computed tomography (CT) scans were observed in 21 of 26 patients.

**Conclusions:**

Cavitary lesions, prior aminoglycoside use, and inappropriate ALIS administration were associated with culture non-conversion. Several factors related to ALIS administration and inhalation management may be modifiable, and appropriate patient education, regular reassessment of inhalation practices, and careful monitoring of ALIS administration may help optimize treatment outcomes.

## Background

The incidence of pulmonary non-tuberculous mycobacterial (NTM) disease has been increasing worldwide [1]. In Japan, both the incidence and mortality of NTM disease now exceed those of tuberculosis [2, 3]. The most commonly isolated organism is *Mycobacterium avium* complex (MAC), which includes *Mycobacterium avium* (*M. avium*) and *Mycobacterium intracellulare* (*M. intracellulare*) [4]. *Mycobacterium avium* complex pulmonary disease (MAC-PD) often shows slow progression with relatively mild symptoms, and therefore watchful waiting is commonly adopted [4]. When patients develop advanced radiologic lesions or symptoms such as hemoptysis, guideline-based therapy (GBT) is initiated with a multidrug antibiotic regimen, and treatment must be continued for at least 12 months after culture conversion [4]. However, some patients have difficulty achieving culture conversion, and overall treatment success rates remain approximately 60% even with GBT [5]. In light of these limitations, amikacin liposome inhalation suspension (ALIS) was approved for refractory MAC-PD by the U.S. Food and Drug Administration in 2018 and in Japan in 2021, raising expectations for improved outcomes. ALIS, a liposomal formulation of amikacin (AMK), enables direct and efficient drug delivery to the pulmonary system, particularly to alveolar macrophages that contain MAC, and reduces systemic toxicity compared with conventional parenteral aminoglycoside administration (e.g., streptomycin [SM] or AMK) [6, 7].

In addition to the phase 3 CONVERT trial published in 2018 [6], several real-world studies have reported the effectiveness and adverse events of ALIS therapy, with culture conversion rates ranging from approximately 30% to 50% [8–10]. At our institution, ALIS has been used for refractory MAC-PD since its approval. During routine clinical practice, we recognized several practical challenges related to ALIS administration, adherence, and inhalation management that appeared to affect treatment effectiveness. Therefore, we conducted a retrospective study to evaluate treatment outcomes, adverse events, and practical factors associated with culture non-conversion in patients treated with ALIS. Based on these findings, we aimed to provide practical considerations for optimizing ALIS therapy in clinical settings.

## Methods

### Study design and definitions.

We retrospectively reviewed all patients with refractory MAC-PD who were treated with ALIS at our institution between October 2021 and June 2025. All patients met the diagnostic criteria for MAC-PD according to the American Thoracic Society (ATS), European Respiratory Society (ERS), European Society of Clinical Microbiology and Infectious Diseases (ESCMID), and Infectious Diseases Society of America (IDSA) [4]. Refractory MAC-PD was defined as MAC-positive sputum culture after ≥ 6 months of GBT, a macrolide-containing multidrug regimen. In this study, at least one MAC-positive sputum culture after ≥ 6 months of GBT was required, and MAC culture positivity was also confirmed in a sputum sample obtained within 3 months before ALIS initiation. This reflects routine clinical practice, in which ALIS was considered and initiated within a relatively short interval after confirmation of culture positivity. These criteria allowed inclusion of patients with only a single documented positive culture after GBT. ALIS was administered only to patients with AMK-susceptible isolates (AMK minimum inhibitory concentration [MIC] ≤ 64 µg/mL) confirmed before ALIS initiation. To assess the effectiveness and adverse effects of ALIS, only patients treated with ALIS for at least 6 months were included in the analysis. The follow-up period extended through December 2025. Baseline clinical characteristics, including age, sex, body mass index (BMI), smoking history, and prior MAC-PD treatment history, were collected from medical records at the time of ALIS initiation. Major comorbidities present at baseline and those newly diagnosed during follow-up were documented. Information on ALIS administration was retrospectively extracted from medical records, including patient self-reports, physician observations, and prescription records, and used to assess the appropriateness of ALIS administration. Sputum samples for acid-fast bacillus (AFB) testing were cultured on 2% Ogawa egg slant medium (Kyokuto, Tokyo, Japan). Isolates were identified as MAC species (*M. avium* or *M. intracellulare*) using polymerase chain reaction (PCR) testing for *Mycobacterium avium* complex (COBAS TaqMan MAI; Roche Diagnostics, Tokyo, Japan). Antimicrobial susceptibility testing was performed with BrothMIC SGM (Kyokuto, Tokyo, Japan). Clarithromycin (CLR) resistance was defined as an MIC ≥ 32 µg/mL, and ALIS resistance was defined as an MIC ≥ 128 µg/mL according to the Clinical and Laboratory Standards Institute (CLSI) M24. Radiological patterns of MAC-PD were classified as noncavitary nodular/bronchiectatic (NC-NB), cavitary nodular/bronchiectatic (C-NB), or fibrocavitary (FC) patterns. Culture conversion was defined as at least three consecutive negative sputum mycobacterial cultures collected at intervals of ≥ 4 weeks after ALIS initiation. The date of culture conversion was defined as the collection date of the first negative sputum sample in the consecutive series. Sputum specimens for mycobacterial culture were collected either spontaneously or by sputum induction with hypertonic saline. Culture non-conversion was defined as persistently positive cultures throughout the treatment period or inability to provide sufficient sputum specimens to confirm three consecutive negative cultures because of incomplete follow-up sputum collection or failure of sputum submission. Isolates from patients with culture non-conversion were retested for the development of acquired CLR or ALIS resistance. Pulmonary fungal disease was diagnosed based on a recent review [11] and the ESCMID/ERS guidelines [12]. The most common form, chronic pulmonary aspergillosis (CPA), was diagnosed based on symptoms lasting > 3 months and characteristic radiologic findings (e.g., fungus ball and pericavitary infiltrates), together with a positive serum *Aspergillus* IgG antibody test, with or without microbiological evidence of *Aspergillus* infection in respiratory samples. Other fungal infections were confirmed by culture of specimens obtained via bronchoscopy. We reviewed ALIS-related adverse effects in light of previous studies and clinical trials showing that patients treated with ALIS may experience various adverse events. Clinical symptoms occurring during treatment and their management were retrospectively collected from medical records, including physician documentation, prescriptions, and medical interventions. Adverse events were graded according to the Common Terminology Criteria for Adverse Events (CTCAE) version 5.0 [13]. For each patient, the highest severity grade observed during ALIS therapy was recorded. Decisions regarding temporary interruption or discontinuation of ALIS were based on both physician judgment and patient self-management in routine clinical practice. Currently, no established diagnostic criteria exist for ALIS-related lung abnormalities. Computed Tomography (CT) scans obtained during ALIS therapy were retrospectively evaluated by the investigators, with reference to radiology reports when available, and compared with CT scans obtained within 3 months prior to ALIS initiation. Follow-up CT examinations during ALIS therapy were performed at the discretion of the treating physicians in routine clinical practice. Radiologic abnormalities were considered potentially ALIS-related based on the following findings: (1) newly developed pulmonary lesions appearing in lung regions distinct from the predominant distribution of pre-existing MAC lesions, including other lobes or the contralateral lung, despite sustained culture negativity after ALIS initiation; (2) development of ground-glass opacity (GGO) or ground-glass attenuation (GGA) consistent with interstitial lung disease, which are atypical radiologic findings for progression of MAC-PD; (3) improvement or partial resolution of the radiologic abnormalities after completion of ALIS therapy; and (4) radiologic progression despite minimal clinical symptoms and absence of significant elevation of inflammatory markers, such as C-reactive protein. Because this study was retrospective and conducted with prior awareness that ALIS-related lung abnormalities may occur, image assessment was not blinded to treatment duration or clinical information. Therefore, radiologic findings were interpreted comprehensively in conjunction with clinical symptoms, inflammatory markers, sputum culture results, treatment course, post-treatment radiologic changes, and the possibility of pulmonary infections other than MAC-PD. Lung abnormalities were categorized as multiple nodular (MN), organizing pneumonia (OP), or hypersensitivity pneumonitis (HP) patterns according to the report by Tokita et al. [10].

### Statistical analyses

Data are presented as numbers and percentages where appropriate. Age, BMI, and serum albumin are expressed as mean ± standard deviation (SD), whereas treatment and follow-up durations are presented as median with interquartile range (IQR) for descriptive statistics. To investigate factors associated with culture conversion, patients were divided into culture conversion and non-conversion groups. Continuous variables were compared using Welch’s t-test (two-sided), and categorical variables were compared using the Chi-square test. Odds ratios (ORs) with 95% confidence intervals (CIs) were calculated for categorical variables. A p-value < 0.05 was considered statistically significant. Statistical analyses were performed using StatMate V software (ATMS, Tokyo, Japan).

### Ethics

This study was conducted in accordance with the Declaration of Helsinki and was approved by the Jichi Medical University Hospital Bioethics Committee for Clinical Research (Approval No. Rindai 24–218). The requirement for informed consent was waived due to the retrospective study design, and patients and their families were given the opportunity to opt out.

## Results

### Patient characteristics

**Fig. 1:**
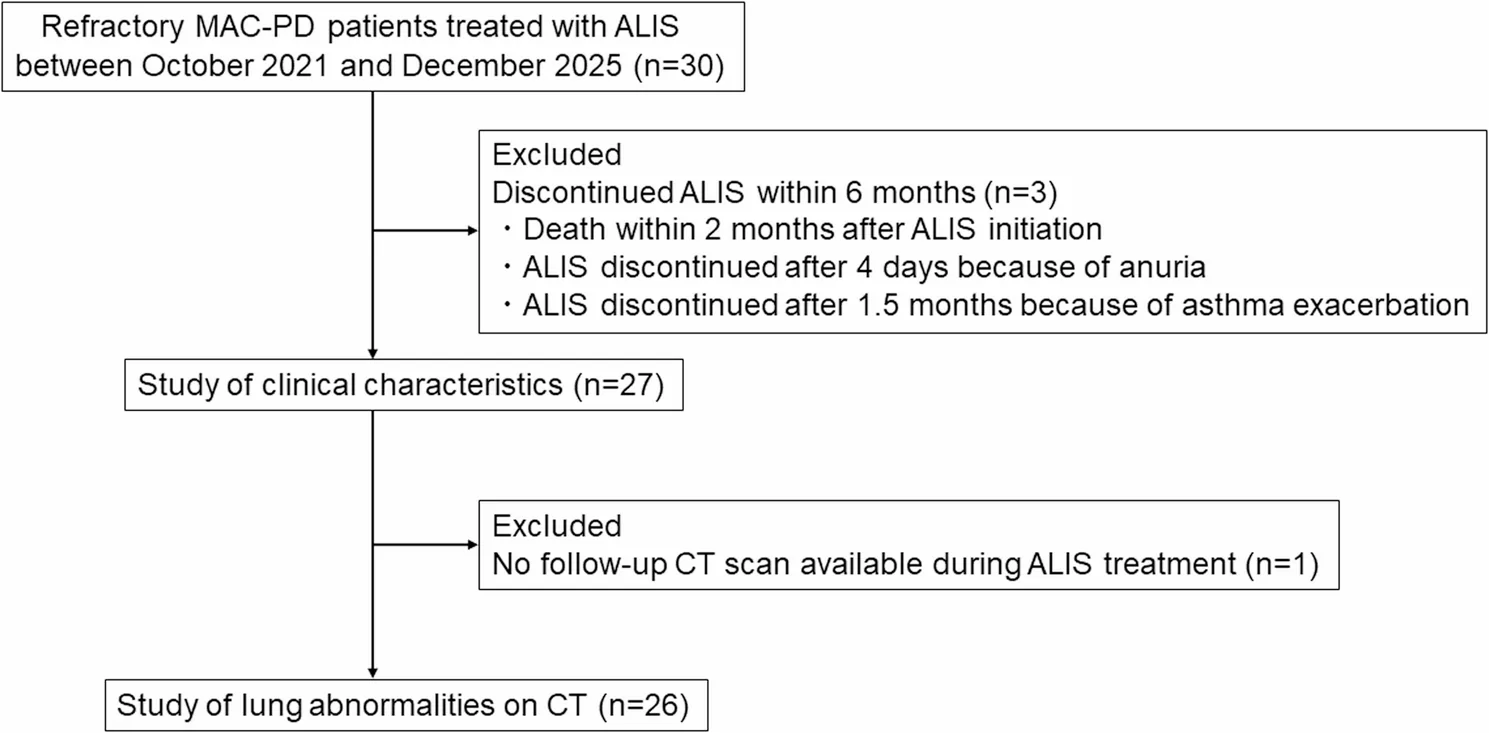
Study flow diagram. Flow diagram showing patient selection and exclusion criteria for the analyses of clinical characteristics and ALIS-related lung abnormalities on computed tomography (CT). ALIS, amikacin liposome inhalation suspension; MAC-PD, *Mycobacterium avium* complex pulmonary disease; CT, computed tomography

Patient characteristics are summarized in Table 1. Of the 30 patients with refractory MAC-PD treated with ALIS during the study period, 3 were excluded because of early discontinuation of ALIS. The reasons were MAC-related death within 2 months after ALIS initiation (*n* = 1), ALIS-associated anuria requiring discontinuation after 4 days of treatment (*n* = 1), and asthma exacerbation leading to discontinuation after 1.5 months of treatment (*n* = 1). Consequently, 27 patients were included in the analysis, all of whom received ALIS for ≥ 6 months (Fig. 1). The mean age was 69.9 ± 6.03 years, and the majority were female (22/27). Approximately half of the patients were underweight (BMI < 18.5 kg/m²), and 6 of 27 were ever-smokers. The most frequent comorbidity was malignancy (9/27), with all but one case in complete remission, followed by pulmonary fungal disease (7/27) and diabetes mellitus (5/27). Among the 7 patients with fungal disease, 6 were diagnosed with CPA. All 6 had positive *Aspergillus* IgG antibodies, and 4 had positive sputum cultures for *Aspergillus*. The remaining patient developed *Scedosporium* infection, diagnosed by bronchoscopy. The most frequently isolated MAC species was *M. avium* (19/27), consistent with regional epidemiology in eastern Japan, where *M. avium* is more prevalent than *M. intracellulare*. Radiological patterns were classified as NC-NB in 12 patients, C-NB in 13, and FC in 2. CLR resistance was identified in 5 patients. No isolates showed ALIS resistance before ALIS initiation. Nine patients had a history of prior aminoglycoside use, including intramuscular SM (*n* = 4), kanamycin (KM) (*n* = 3), and intravenous AMK (*n* = 2). All patients had received GBT prior to ALIS initiation. Baseline GBT regimens at the time of ALIS initiation included 3 drugs in 14 patients, 2 drugs in 11 patients, and 4 drugs in 1 patient. One patient was receiving ALIS monotherapy at baseline due to intolerance to oral therapy; however, this patient had previously received a three-drug regimen prior to ALIS initiation and resumed combination therapy 6 months after ALIS initiation. Seven patients received regimens without ethambutol (EB), including 5 who had previously developed optic neuropathy and 2 who declined EB due to concerns about visual toxicity. The median duration of ALIS therapy was 14.6 months (IQR, 7.96–23.6), and the median follow-up period was 23.0 months (IQR, 12.0-30.1). Spontaneous sputum samples were obtained in 22 patients, whereas sputum induction with hypertonic saline was required in 5 patients. One patient was unable to provide three consecutive sputum samples for assessment of culture conversion. Only two sputum cultures were obtained in this patient, and the second culture remained positive for MAC. Because sputum production had also not decreased, this patient was considered unlikely to have achieved culture conversion and was therefore classified as non-conversion. Consequently, the sputum culture conversion rate was 51.8% (14/27; 95% CI, 34.0–69.3%). Among the 13 patients with non-conversion, newly acquired CLR resistance developed in 3 patients and ALIS resistance developed in 1 patient. During the follow-up period, 4 patients died, including 2 deaths attributable to MAC progression.

**Table 1:** Clinical characteristics of patients treated with ALIS

Total patients treated with ALIS, *n*	30
Early discontinuation of ALIS within 6 months, n (%)	3 (10%)
Reason for early discontinuation	
Death, n	1
ALIS-associated anuria, n	1
Asthma exacerbation, n	1
Patients treated with ALIS ≥ 6 months, n (%)	27 (90%)
Age, years, mean ± SD	69.9 ± 6.03
Sex, Male/Female	5/22
BMI, kg/m^2^, mean ± SD	18.1 ± 2.76
Ever smokers, n/N	6/27
Comorbidities	
Malignancy, n/N	9/27
Pulmonary fungal disease, n/N	7/27
Diabetes, n/N	5/27
Collagen disease, n/N	3/27
MAC species	
* M. avium*, n/N	19/27
* M. intracellulare*, n/N	8/27
Cavitary lesions, n/N	15/27
Radiological patterns	
NC-NB, n/N	12/27
C-NB, n/N	13/27
FC, n/N	2/27
CLR resistance, n/N	5/27
Prior aminoglycoside use, n/N	9/27
Number of GBT drugs at baseline, 0/2/3/4, n	1/11/14/1
Regimen at baseline, n/N	
EB/Macrolide/RFP/Other	1/27
EB/Macrolide/RFP	13/27
Macrolide/RFP/Other	1/27
EB/Macrolide	6/27
Macrolide/Other	5/27
Treatment duration with ALIS, months, median (IQR)	14.6 (7.96–23.6)
Follow-up period, months, median (IQR)	23.0 (12.0-30.1)
Outcomes	
Culture conversion, n/N (%) [95% CI]	14/27 (51.8%) [34.0-69.3]
Newly developed CLR resistance, n/N	3/27
Newly developed ALIS resistance, n/N	1/27
Death, n/N	4/27
MAC-related, n/N	2/27
Non-MAC-related, n/N	2/27

Data are presented as n/N, where n is the number of patients with the characteristic and N is the total number of analyzed patients (*N* = 27)

*ALIS *amikacin liposome inhalation suspension, *SD *standard deviation, *BMI *body mass index, *MAC* *Mycobacterium avium* complex; *NC-NB *noncavitary nodular bronchiectatic, *C-NB *cavitary nodular bronchiectatic, *FC *fibrocavitary, *CLR *clarithromycin, *GBT *guideline-based therapy, *EB *ethambutol, *RFP *rifampicin, *IQR *interquartile range, *CI* confidence interval

### Factors associated with culture conversion

Patients were divided into culture conversion and non-conversion groups to identify factors associated with treatment efficacy. As shown in Table 2, age, sex, BMI, serum albumin level, and treatment duration did not significantly differ between the 2 groups. In contrast, cavitary lesions, prior aminoglycoside use, and inappropriate ALIS administration were significantly associated with culture non-conversion. Pulmonary fungal disease, baseline CLR resistance, and non-EB regimens were not significantly associated with culture conversion.

**Table 2:** Comparison of culture conversion and non-conversion groups

	Culture conversion (*n* = 14)	Non-conversion (*n* = 13)	OR (95% CI)	*P*-value
Age, years, mean ± SD	68.6 ± 7.31	71.4 ± 4.09		0.24^a^
Sex, Male/Female	3/11	2/11	1.5 (0.21–10.8)	0.68^b^
BMI, kg/m^2^, mean ± SD	18.7 ± 2.77	17.5 ± 2.74		0.29^a^
Smoking history, n	4	2	2.2 (0.33–14.7)	0.41^b^
Albumin, g/dL, mean ± SD	4.07 ± 0.44	3.94 ± 0.46		0.48^a^
Treatment duration with ALIS, months, mean ± SD	18.7 ± 9.63	15.9 ± 10.1		0.46^a^
MAC species *M. avium*/*M. intracellulare*	8/6	11/2	0.24 (0.04–1.53)	0.12^b^
Diabetes, n	3	2	1.5 (0.21–10.8)	0.68^b^
Pulmonary fungal disease, n	3	4	0.61 (0.11–3.49)	0.58^b^
Cavity, n	5	10	0.17 (0.03–0.90)	0.03^b^
Prior aminoglycoside use, n	2	7	0.14 (0.02–0.91)	0.029^b^
CLR resistance, n	3	2	1.5 (0.21–10.8)	0.68^b^
Non-EB regimen, n	4	3	1.33 (0.24–7.56)	0.74^b^
Inappropriate use of ALIS, n	1	7	0.07 (0.007–0.66)	0.0079^b^

*SD *standard deviation, *BMI *body mass index, *MAC* *Mycobacterium avium* complex, *CLR *clarithromycin, *EB *ethambutol, *ALIS *amikacin liposome inhalation suspension, *OR *odds ratio, *CI *confidence interval

^a^ Welch’s test (two-sided)

^b^ Chi-square test

### Details of inappropriate ALIS administration

As noted above, 8 patients (approximately 30%) were identified as having used ALIS inappropriately (Table 3), of whom 7 did not achieve culture conversion. Based on the definition of prolonged intermittent ALIS use reported by Kurahara et al. [9], 4 patients received < 80% of the expected number of ALIS vials for > 3 months, with actual prescription rates ranging from approximately 50% to 70%. Three patients declined daily inhalation because of adverse effects such as ALIS-induced dysphonia, resulting in prolonged intermittent use. One patient occasionally interrupted ALIS without medical consultation and did not adequately shake and resuspend the suspension prior to inhalation, resulting in approximately a third of the drug remaining in the vial after nebulization. In addition, nebulizer handset replacement was inadequate in 2 patients, with only approximately 60% of the expected number of handsets prescribed during treatment because only 1 handset was prescribed for every 2 months of ALIS therapy. In another patient, nasal breathing during nebulization was reported for approximately 20% of the ALIS treatment period, suggesting insufficient drug delivery to the lower respiratory tract. Lastly, 1 patient received ALIS monotherapy for 6 months.

**Table 3:** Details of inappropriate ALIS administration

Details of inappropriate use of ALIS (*n* = 8)	*n*
Long-term (> 3 months) intermittent use (< 80% of expected ALIS vials)	3
Long-term (> 3 months) intermittent use (< 80% of expected ALIS vials) and insufficient resuspension	1
Inadequate replacement of the nebulizer handset	2
Nasal breathing instead of oral inhalation	1
ALIS monotherapy without concomitant GBT	1

*ALIS *amikacin liposome inhalation suspension, *GBT *guideline-based therapy

### Adverse effects and management

The most frequently observed adverse event was dysphonia (21/27), typically developing within 1–2 months after initiation, followed by cough (16/27). Other adverse events included increased sputum production, oral pain, and hemoptysis (each 4/27), tinnitus (3/27), sore throat (2/27), pneumothorax (1/27), and fever (1/27). Most adverse events were Grade 1 or 2 and were managed medically or with supportive care (Table 4). In contrast to the prolonged intermittent use observed in some patients with dysphonia, temporary interruption because of other adverse events, including tinnitus and sore throat, was generally followed by resumption of daily inhalation within 3 months. Two patients developed late complications requiring permanent discontinuation of ALIS. One patient developed severe hemoptysis 7 months after ALIS initiation and required emergency hospitalization and bronchial artery embolization. This patient had underlying bronchiectatic disease, a large cavitary lesion > 40 mm in diameter, and concomitant pulmonary *Aspergillus* infection, all of which were considered possible contributing factors. Although ALIS was resumed after discharge, recurrent hemoptysis developed thereafter, leading to permanent discontinuation of ALIS. No recurrent hemoptysis was observed after discontinuation. Another patient developed severe pneumothorax 2 years after ALIS initiation and required hospitalization with chest drainage. This patient had advanced FC pattern with multiple cavitary lesions, and rupture of a cavitary lesion was considered the most likely cause. One week after chest drainage, thoracoscopic left lower lobectomy and lingulectomy were performed. Because persistent cough associated with inhalation therapy was considered a potential risk factor for recurrent pneumothorax, ALIS was permanently discontinued.

**Table 4:** Adverse effects of ALIS and management

Adverse effects	Patients	Management	No. of patients
Dysphonia	21/27		
Grade 1 or Grade 2	19/21	Gargling and/or dequalinium chloride	12/21
		Temporary discontinuation and intermittent use of ALIS	3/21
		None	4/21
Grade 3	2/21	Gargling and dequalinium chloride	2/21
Cough	16/27		
Grade 1	10/16	Antitussives	1/16
		None	9/16
Grade 2	6/16	Short-acting β2-agonist inhalation	1/16
		ICS/LABA	5/16
Increased sputum production	4/27		
Grade 1 or Grade 2	4/4	None	4/4
Oral pain	4/27		
Grade 2	4/4	Gargling with antifungal agents	2/4
		Oral care	1/4
		Topical oral corticosteroid	1/4
Hemoptysis	4/27		
Grade 1	1/4	None	1/4
Grade 2	2/4	Tranexamic acid	2/4
Grade 4	1/4	BAE	1/4
Tinnitus	3/27		
Grade 1	2/3	None	2/3
Grade 2	1/3	Temporary discontinuation and intermittent use of ALIS	1/3
Sore throat	2/27		
Grade 2	2/2	Dequalinium chloride	1/2
		Temporary discontinuation and intermittent use of ALIS	1/2
Pneumothorax	1/27		
Grade 3	1/1	Chest drainage and subsequent lobectomy	1/1
Fever	1/27		
Grade 1	1/1	Antipyretics	1/1
ALIS-related lung abnormalities	21/26		
MN pattern	16/21		
MN + OP pattern	4/21		
HP pattern	1/21		

Adverse effects were graded according to the Common Terminology Criteria for Adverse Events (CTCAE) version 5.0. Denominators indicate the relevant study population for each row. For severity grades and management, denominators represent the number of patients who developed the corresponding adverse event

ALIS, amikacin liposome inhalation suspension; ICS/LABA, inhaled corticosteroids and long-acting β2-agonists; BAE, bronchial artery embolization; MN, multiple nodular; OP, organizing pneumonia; HP, hypersensitivity pneumonitis

### ALIS-related lung abnormalities

**Fig. 2:**
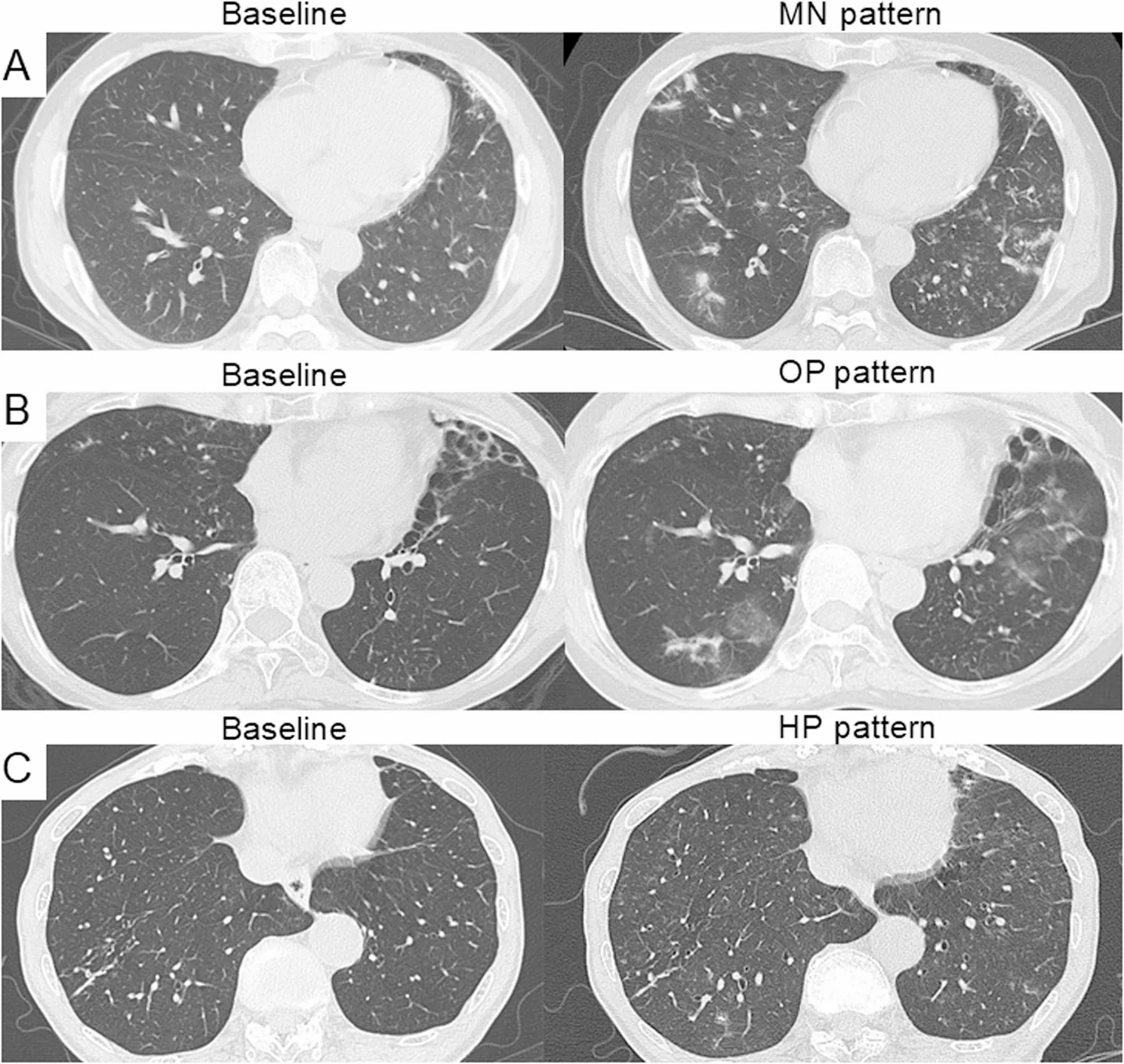
CT findings before ALIS therapy (baseline) and after the development of ALIS-related lung abnormalities. **A **Baseline (left) and multiple centrilobular nodules (right, MN pattern). **B **Baseline (left) and patchy ground-glass opacities with a reversed halo sign (right, OP pattern). **C **Baseline (left) and diffuse ill-defined centrilobular micronodules (right, HP pattern). CT, computed tomography; ALIS, amikacin liposome inhalation suspension; MN, multiple nodular; OP, organizing pneumonia; HP, hypersensitivity pneumonitis

ALIS-related lung abnormalities on CT were observed in this study. Follow-up CT scans were available for 26 of 27 patients (Fig. 1), and 21 of 26 developed radiologic abnormalities. Among the 21 patients with ALIS-related lung abnormalities, MN patterns (Fig. 2A) were observed in 16, MN and OP patterns (Fig. 2B) in 4, and an HP pattern (Fig. 2C) in 1 (Table 4). Most patients were asymptomatic or had only mild sputum production and mild cough, and no additional treatment was required. One patient with an HP pattern developed moderate cough and mild dyspnea 6 weeks after ALIS initiation but improved with ICS/LABA therapy without discontinuation of ALIS. Two patients with MN patterns discontinued ALIS because the radiologic findings were initially interpreted as MAC progression; however, the abnormalities resolved after ALIS discontinuation.

## Discussion

In the present study, we identified several important considerations for achieving culture conversion in patients with refractory MAC-PD treated with ALIS. Here, we discuss key points for optimizing ALIS therapy.

Our analyses showed that cavitary lesions and prior aminoglycoside use were significantly associated with failure to achieve culture conversion. Previous studies of ALIS, including Japanese real-world cohorts, have also reported lower conversion rates in patients with cavitary lesions than in non-cavitary cases [8–10]. Consistent with these findings, the recent Amikacin liposome inhalation suspension in newly diagnosed *Mycobacterium avium* complex lung disease (ARISE) trial demonstrated favorable microbiological outcomes in patients with newly diagnosed or recurrent noncavitary MAC-PD treated with ALIS-containing therapy [14]. Taken together with our findings, these observations suggest that the presence of cavitary lesions may substantially influence treatment response to ALIS. Cavitary lesions are considered to contain a high mycobacterial burden [15] and may limit drug penetration. For this reason, parenteral aminoglycosides are often administered during the initial phase of treatment in addition to oral GBT. However, the duration of parenteral aminoglycoside therapy is generally limited to 3–6 months because of irreversible adverse effects such as ototoxicity. In contrast, ALIS allows prolonged inhaled aminoglycoside administration, sometimes exceeding 1–2 years, as shown in previous studies [9, 10] and in our cohort. Although conversion rates were lower in cavitary cases, 5 of 15 patients with cavitary lesions in our study achieved culture conversion, suggesting that ALIS may provide therapeutic benefit even in this high-risk population. Another important consideration is the potential role of surgical resection in patients with localized cavitary disease and persistent culture positivity despite appropriate medical therapy, as drug penetration into cavitary lesions may be limited [16]. In fact, 1 patient with persistently positive cultures underwent surgical resection 2 months after the study period ended.

As shown in Table 3, approximately 30% of patients demonstrated inappropriate ALIS administration, which was significantly associated with culture non-conversion. These findings suggest that proper administration technique and regular monitoring may be important for optimizing ALIS therapy in routine clinical practice. First, intermittent inhalation used for managing adverse effects may result in prolonged non-daily ALIS use and reduced adherence, potentially affecting adherence to concomitant oral GBT as well. Furthermore, suboptimal adherence may be overlooked, as patients may not readily report deviations from prescribed regimens. Given the high cost of ALIS, some patients may intentionally reduce dosing frequency, potentially compromising efficacy. Second, adequate resuspension of ALIS prior to inhalation is essential. The medication guide recommends removing the vial from refrigeration at least 45 min before use [17]; however, inadequate equilibration to room temperature or insufficient mixing before inhalation can lead to incomplete nebulization and reduced drug delivery. In colder climates, a longer equilibration time may be necessary to ensure adequate resuspension before nebulization. Third, the nebulizer handset should be replaced monthly [17]. Inadequate replacement of the handset may reduce nebulization efficiency, as failure to replace the device regularly can lead to clogging of the fine pores in the metal aerosol head. Clinicians should also ensure that patients disassemble and disinfect the handset daily and clean the metal aerosol head with an ultrasonic cleaning device on a weekly basis. Fourth, proper oral inhalation technique is essential to ensure adequate pulmonary drug delivery. Periodic reassessment of inhalation technique may therefore be necessary. Fifth, appropriate combination therapy with ALIS should be maintained, and monotherapy should be avoided to minimize the risk of drug resistance development [18]. Given the limited effective treatment options currently available for MAC, regimens consisting of only 2 drugs, such as ALIS plus a macrolide, should be used with caution, as interruption of ALIS may effectively result in macrolide monotherapy. Lastly, at our institution, nurses primarily confirmed inhalation technique at ALIS initiation and the subsequent follow-up visit, whereas subsequent assessments largely depended on patient self-report and physician observation in routine clinical practice. Because no standardized institutional protocol for long-term inhalation monitoring was in place, recognition of inappropriate ALIS administration may have been delayed in some patients. These findings highlight the importance of continuous education and regular reassessment of inhalation technique during ALIS therapy. Nevertheless, given the retrospective design of this study, causality cannot be established. In addition, some deviations from the prescribed ALIS regimen may have been related to adverse events.

Previous reports have demonstrated that adverse events are relatively common during ALIS therapy and may contribute to treatment discontinuation [6, 8–10]. Dysphonia and cough are the most frequently reported adverse effects and can be uncomfortable for patients. In both a clinical trial [6] and our cohort, most of these adverse effects resolved within approximately 2 months. Therefore, intermittent inhalation should be temporary whenever possible. When additional management is required to maintain daily inhalation, dysphonia may be alleviated by frequent gargling and/or dequalinium chloride. In Japan, pre-treatment inhalation of a β2-agonist has been recommended for ALIS-related cough [19], and in selected cases, inhaled corticosteroids and long-acting β2-agonists (ICS/LABA) therapy may provide more rapid symptom control. Although inhaled corticosteroids are theoretically associated with an increased risk of infection progression [20], their use in our cohort was limited and did not appear to adversely affect disease control. Oral pain was also observed in some ALIS users and may discourage continued use. Two patients developed candidal stomatitis, which resolved within 2 months after treatment with an amphotericin B-diluted gargle. In addition, 1 patient developed black hairy tongue, which was managed with oral hygiene measures and may have been associated with prolonged antibiotic therapy. Because such oral complications may negatively affect patients’ willingness to continue ALIS, careful assessment of oral conditions and appropriate management are important to maintain treatment adherence. Notably, among the 3 patients who discontinued ALIS within 6 months and were excluded from the final analysis, 2 discontinued treatment because of serious adverse events. One patient developed anuria due to severe aminoglycoside-associated nephrotoxicity, which required immediate discontinuation of ALIS. Another patient experienced an asthma exacerbation after ALIS initiation despite previously stable disease control, resulting in ALIS discontinuation. Bronchospasm and nephrotoxicity are recognized adverse effects of ALIS [18] and may lead to treatment discontinuation. Therefore, careful monitoring for respiratory and renal complications is warranted during ALIS therapy. Radiologic abnormalities have also been reported in patients receiving ALIS [10, 21, 22]. The most frequent pattern is the MN pattern, which may be misinterpreted as radiologic worsening of NTM-PD, potentially leading to unnecessary discontinuation of ALIS. Compared with true NTM progression, ALIS-related nodules on CT tend to be distributed predominantly in the lower lobes and may appear outside the primary NTM lesions. ALIS consists of amikacin sulfate encapsulated in liposomes composed of phospholipids and cholesterol, which may contribute to the development of lipoid pneumonia-like changes. Furthermore, even when ALIS-related lung abnormalities are observed, most patients do not exhibit significant clinical symptoms [10]. Therefore, to ensure appropriate continuation of ALIS therapy, clinicians should carefully interpret radiologic findings in conjunction with clinical symptoms and sputum examination results.

In this study, 7 of 27 patients had pulmonary fungal disease, most commonly *Aspergillus* infection. Although no statistically significant association was observed between fungal disease and culture conversion, the prevalence in our cohort was notably higher than previously reported [23, 24], with most (5 of 7) cases newly diagnosed after ALIS initiation. Previous studies have shown that CPA development is associated with both smear-positive AFB [25] and failure to achieve culture conversion [23]. Poorer survival has also been observed in patients with CPA and concomitant NTM infection [26]. Therefore, careful monitoring for fungal disease, including *Aspergillus* IgG antibody testing and radiologic follow-up, may be considered in patients with cavitary lesions or bronchiectasis during ALIS therapy.

This study has several limitations. First, this was a retrospective single-center study with a relatively small sample size. Given the limited number of outcome events, multivariable analyses were not performed owing to concerns regarding overfitting and unstable estimates, and residual confounding may remain. In addition, multiple comparisons were performed in this exploratory analysis, and the possibility of Type I errors should be considered. This cohort also reflected the regional epidemiology of eastern Japan, where *M. avium* is more prevalent than *M. intracellulare*, potentially limiting the generalizability of these findings to other geographic regions or MAC-PD populations. Nevertheless, the early discontinuation rate was low (10%), and all patients were followed throughout the study period. As this study reflects real-world clinical practice over a prolonged period, it provides insight into both the effectiveness of ALIS and the practical challenges associated with its use. Moreover, follow-up CT was available for nearly all patients, allowing detailed evaluation of ALIS-related lung abnormalities.

## Conclusions

In conclusion, addressing the challenges related to ALIS use identified in this study may help optimize ALIS therapy. As in asthma management, ALIS is an inhaled medication that requires proper technique. Therefore, both patients and clinicians should clearly understand the correct administration technique and ensure appropriate inhalation to optimize treatment efficacy.

## Data Availability

The datasets used and analyzed during the current study are available from the corresponding author on reasonable request.

## Abbreviations

ALIS: Amikacin liposome inhalation suspension
NTM: Non-tuberculous mycobacteria
MAC: Mycobacterium avium complex
M. avium: Mycobacterium avium
M. intracellulare: Mycobacterium intracellulare
MAC-PD: Mycobacterium avium complex pulmonary disease
GBT: Guideline-based therapy
AMK: Amikacin
SM: Streptomycin
ATS: American Thoracic Society
ERS: European Respiratory Society
ESCMID: European Society of Clinical Microbiology and Infectious Diseases
IDSA: Infectious Diseases Society of America
MIC: Minimum inhibitory concentration
BMI: Body mass index
AFB: Acid-fast bacillus
PCR: Polymerase chain reaction
CLR: Clarithromycin
EB: Ethambutol
CLSI: Clinical and Laboratory Standards Institute
NC-NB: Nodular/bronchiectatic
C-NB: Cavitary nodular/bronchiectatic
FC: Fibrocavitary
CPA: Chronic pulmonary aspergillosis
CTCAE: Common Terminology Criteria for Adverse Events
CT: Computed tomography
Ground-glass opacity: GGO
Ground-glass attenuation: GGA
MN: Multiple nodular
OP: Organizing pneumonia
HP: Hypersensitivity pneumonitis
SD: Standard deviation
IQR: Interquartile range
Odds ratios: ORs
Confidence intervals: CIs
Kanamycin: KM
ICS/LABA: Inhaled corticosteroids and long-acting β2-agonists
